# Validation of a measure of the Satter eating competence model with low-income females

**DOI:** 10.1186/1479-5868-8-26

**Published:** 2011-04-07

**Authors:** Jodi S Krall, Barbara Lohse

**Affiliations:** 1 Weight Management and Wellness Center, University of Pittsburgh Medical Center, Pittsburgh, PA, USA; 2 Department of Nutritional Sciences, The Pennsylvania State University, University Park, PA, USA

## Abstract

**Background:**

The purpose of this study was to evaluate the construct validity of a version of the ecSatter Inventory (ecSI), a measure of eating competence (EC), as adapted for use in a low-income (LI) population.

**Methods:**

Females (n = 507), aged 18 to 45 years, living in households with a history of participating in the Supplemental Nutrition Assistance Program completed a web-based survey that included the ecSI for LI (ecSI/LI) and valid measures of cognitive and affective eating behavior, food preference and practice, and food preparation.

**Results:**

Most correlations and differences between eating competent and non-eating competent categories and among EC tertiles were compatible with hypothesized relationships. ecSI/LI scores were positively related with self-reported physical activity, food acceptance, fruit and vegetable intake, and food planning/resource management. ecSI/LI scores were negatively associated with body mass index, dissatisfaction with body weight, tendency to overeat in response to external or emotional stimuli, and indices of psychosocial attributes related to disordered eating.

**Conclusions:**

The ecSI/LI is a valid measure of EC for low-income females and provides a tool for researchers and educators to assess intervention outcomes and further explore the EC construct.

## Background

Socioeconomic status (SES) is associated with diet quality and related health outcomes. Data suggest that low-income groups have higher intakes of energy-dense foods and lower intakes of fruits, vegetables, and fiber [1-4], a dietary pattern known to be associated with poorer health outcomes [3,5]. In particular, low-income women consistently express higher rates of obesity [6,7] and type II diabetes [8,9] compared to women from higher income groups. However, the nature of the relationship between SES, diet quality, and health disparities remains largely unexplained.

Hypotheses derived from literature suggest that the relatively poorer dietary behaviors of low-income populations may be the result of environmental, social, and individual influences. Cognitive and affective dimensions of eating behaviors specific to low-income individuals are not conducive to successful navigation of the current food environment. Compared to women of higher SES, women of lower SES are more likely to base food choices on familiarity and price [10] and less likely to place importance on health considerations [11,12] or nutrition knowledge and beliefs [13,14]. In addition, low-income adults, and, in particular, low-income mothers, spend less time preparing meals and rely more heavily on ready-to-eat meals than individuals with higher SES [12,15-17]. Furthermore, women of lower SES exhibit less restrictive dietary practices [18], are less concerned about weight [19], and are less likely to try to lose weight [18,19] than women of higher SES.

Behavioral models are needed to approach the understanding of the modifiable determinants of food choices among low-income populations. The Satter Model of Eating Competence (ecSatter), an emerging model of eating behavior that addresses intrapersonal approaches to eating and food-related behaviors, may be an appropriate model for understanding and improving diet-related health of low-income groups [20]. An evidence- and practice-based model, ecSatter is the outcome of repeated clinical observations of distorted eating attitudes and behaviors and identification of modifiable factors that facilitate resolution of those distortions [20]. ecSatter encompasses four interrelated components: 1) attitudes about eating and enjoyment of food, 2) food acceptance skills that support dietary variety, 3) internal regulation skills that address energy balance, and 4) skills and resources for managing food selection, meal preparation, and mealtime structure. The successful adoption of these four ecSatter components results in *eating competence *(EC). Research with diverse samples indicates that EC is positively associated with healthy eating patterns [21-23] and healthier profiles of cardiovascular disease risk factors [21,24]. Moreover, qualitative assessment of EC in a low-income sample suggests that ecSatter provides a tenable framework for rationalizing low-income individuals' cognitive and affective responses to eating experiences [25].

EC is measured with the ecSatter Inventory (ecSI), a reliable instrument [26] for which construct dimensionality and validity were established with a general sample of men and women (n = 863; mean age 36.2 ± 13.4 years, range = 18 to 71 years) [22]. To evaluate the appropriateness of the instrument for a low-income audience, the ecSI was subjected to cognitive testing with a sample of 25 low-income women (18 to 49 years old) [27]. Four of the 16 ecSI items were misinterpreted for various reasons, including problems with clarity and wording. The misinterpreted items were revised based on participant feedback, retested, and combined with the 12 unaltered items into a new instrument, termed the ecSI for Low-Income (ecSI/LI). The ecSI/LI is comparable to the ecSI in content and scope and requires large scale validation. The objective of the present study was to confirm construct validity of ecSatter in a low-income population using a concurrent validity approach.

## Methods

### Recruitment

The Pennsylvania Department of Public Welfare generated a database of 40,080 households participating in Supplemental Nutrition Assistance Program (SNAP) as of March 2007. These households included children under 18 years and were located in SNAP-Education (SNAP-ED)-served counties. Invitational letters (n = 1000) and post-cards (n = 16,050) delineating study details and survey access instructions were mailed in 2 waves to 17,050 households randomly selected from the aforementioned database.

Eligible respondents were female, members of the randomly selected SNAP households, 18 to 45 years of age, literate in English, with internet access; only 1 female per household was allowed to participate. Exclusion criteria included full-time enrollment at a four-year college or university, studying to become or employed as a nutritionist, or a diet-related health issue (i.e., diagnosis of diabetes, cancer, or heart, liver or lung disease in the past five years or a recipient of surgery for weight loss). This study was approved by the Pennsylvania State University Institutional Review Board.

### Research Design and Data Collection

The research design was cross-sectional with concurrent data collection using the Internet. Invitations directed potential participants to a web-based survey that detailed study procedures and included an informed consent. Eligibility verification was required. Potential participants answered specific eligibility questions; an ineligibility notice was sent at the point at which a criterion was not met. Meeting all eligibility criteria prompted informed consent. Eligible individuals who agreed to participate were given survey access; completion prompted mailing of a $10 gift card. This survey access process was pilot tested for feasibility with 5 participants from a local health clinic serving the target audience. Average survey completion time approximated 45 minutes.

The active survey website was accessed by 635 individuals. Of these individuals, 38 were ineligible to participate (30 for health reasons, three were full-time students, and two were nutrition-related professionals or students); five were not interested in completing the survey after reading a more thorough description of the study process; 67 started but did not complete the survey; 18 provided unusable responses on the completed survey. In all, 507 usable surveys were collected.

### Measures

The web-based survey consisted of the ecSI/LI as well as instruments and items to assess the validity of the ecSI/LI and to gather information about respondent sociodemographic characteristics. Survey item order was identical for all respondents with the exception of the ecSI/LI; half of respondents completed the ecSI/LI at the beginning of the survey and the other half completed it at the end. Completion order was randomly assigned.

#### ecSatter Inventory for Low-Income (ecSI/LI)

The ecSI/LI consists of 16 Likert-scaled items with 5 response options (never = 0, rarely = 0, sometimes = 1, often = 2, and always = 3) summed to yield a total score (range: 0-48) and four subscale scores: Eating Attitudes (0-15), Food Acceptance (0-9), Internal Regulation (0-9), and Contextual Skills (0-15). ecSI/LI total scores of 32 or higher indicate EC [22]. ecSI/LI items were previously developed and tested with low-income adults [22,27]. The ecSI/LI is available at http://www.ellynsatter.com.

#### Three-Factor Eating Questionnaire (TFEQ)

The validated 51-item TFEQ measures cognitive restraint, disinhibition, and hunger dimensions of eating behavior [28]. The TFEQ includes 15 Likert-scaled items and 36 true/false items. Higher scores on the respective scales are indicative of greater cognitive restraint (conscious restriction of food intake to prevent weight gain or promote weight loss; range: 0-16), disinhibition (tendency to overeat in response to various stimuli such as emotional distress; range: 0-21), and hunger (subjective feeling of hunger and food cravings; range: 0-14).

#### Eating Disorders Inventory-3 (EDI-3)

The EDI-3, a measure of symptoms commonly associated with anorexia nervosa and bulimia, is a validated questionnaire consisting of 12 subscales derived from 92 items using a 6-point Likert scale [29]. Drive for thinness, bulimia, and body dissatisfaction are three scales that measure attitudes and behaviors concerning eating, weight and body shape, specifically fear of fatness, thinking about and practicing bingeing, and dissatisfaction with overall shape, respectively. The remaining nine scales assess psychological domains of disordered eating: low self-esteem and personal alienation scales measure self-evaluation and sense of emotional emptiness; interpersonal insecurity and interpersonal alienation indicate individuals' beliefs about social relationships; interoceptive deficits and emotional dysregulation reflect ways that individuals interpret and respond to emotional cues; perfectionism and asceticism reflect the pursuit of perfection through self-denial and suffering; and maturity fears indicates desire to retreat to the security of childhood. For all scales, the higher the score, the stronger the endorsement of the eating disorder domain. For this study, raw scores were calculated and converted to percentile ranks using United States adult clinical sample tables, as described by Garner [29].

#### Food preference survey

Food preference has been shown to be a proxy for food intake [30,31]. The survey includes 62 food items scored on a 9-point spread semantic differential scale anchored by *dislike extremely *and *like extremely *with separate choices for *never tried *and *would not try*. For this study, item scores were summated to create a total preference score; a separate score for never tried/would not try was also calculated. Preference responses were dichotomized into like (≥5) and dislike (≤4) categories. Discriminating preference patterns were achieved by summing preference scores for like and dislike categories and dividing each by the number of food items in each category to create Food Like and Food Dislike Indices [22]. Preference scores for disliked foods were reversed to compute the dislike index enabling unidirectional interpretation of both indices.

#### Fruit and vegetable stage of change algorithm

Stages of Change (i.e. precontemplation, contemplation, preparation, action, and, maintenance) [32] for fruit and vegetable intakes were measured separately with a validated, two-step algorithm [33]. Fruit and vegetable intake was queried as well as duration of and intention to increase intake. These queries were used to determine meeting recommended levels of intake as well as stage placement. Prior to analysis, stages of change were collapsed into two categories - pre-action (precontemplation, contemplation, and preparation) and action/maintenance.

#### Expanded Food and Nutrition Education Program (EFNEP) questions

Questions about food resource management, preparation, and practices, and health locus of control were derived from the EFNEP Evaluation and Reporting System, a valid and reliable system widely tested with low-income populations [34]. Sixteen items were answered using a 5-point Likert scale. An additional five items, with 3-point Likert scales, are summed to derive health locus of control, an individual's perceived ability to control health-related outcomes. For all items and the health locus of control scale, the higher the score, the stronger the endorsement of the item or scale. Food preparation and practice was additionally assessed with two non-EFNEP items - "*How often do you prepare food at home?*" and "*How do you feel about cooking?*"

#### United States (US) Adult Food Security Survey Module

Developed by the US Department of Agriculture, this 10-item scale assesses food security of adults at the household level [35]. The sum of affirmative responses to the items provides a household's raw score on the scale (possible range of 0-10). From raw scores, food security status among adults is assigned as high (raw score = 0), marginal (1-2), low (3-5), and very low (6-10). "*Don't know*" is included as a possible response option for each item; food security scores were not calculated for participants who chose this response option.

#### Additional items

The survey also contained sociodemographic items, including SNAP use and worry about money for food ("*Do you ever worry about not having enough money to buy food*?"). Self-reported height and weight were used to calculate body mass index (BMI) and physical activity was assessed by asking "*Do you consider yourself a physically active person*?". Level of weight satisfaction ("*How satisfied are you with your current weight*?") was assessed with a 5-point Likert scale (1 = very satisfied, 5 = very unsatisfied).

### Data Analysis

Data were analyzed with SPSS (version 16.0 for Windows, SPSS, Inc., Chicago, IL). Descriptive statistics, including measures of central tendency and distribution, were calculated to describe the sample as well as ecSI/LI total and subscale scores. Internal consistency of the ecSI/LI composite scale and subscales was assessed by Cronbach's alpha [36]. Dichotomized ecSI/LI scores were compared to continuous and categorical variables using t-tests and chi-square tests, respectively. Analysis of variance with post hoc analyses using Bonferroni corrections and chi-square analyses were used to compare descriptive variables and measures of eating attitudes and behaviors across ecSI/LI tertiles. Concurrent validity was also assessed with Pearson product moment correlations between ecSI/LI scores and previously described measures of eating attitudes and behavior. Correlations were evaluated based on *a priori *hypotheses derived from concurrent validation of the ecSI [22]. Correlations < ± .20 were interpreted as not associated [37].

## Results

### Respondent Characteristics

Respondents (n = 507; 100% female) were mostly white (78%), non-Hispanic (93.9%) mothers (96.6%) with a mean age of 31.81 ± 7.0 years (Table 1). Education experience varied, with high school completion the most frequently selected level (35.3%). At the time of the study, 86% of respondents reported living in households participating in SNAP. Food security status varied, with the highest percentage of respondents living in households with very low food security among adults. Based on self-reports, about half of the sample was physically active; mean BMI was 30.41 ± 8.6. Respondents had sole or shared responsibility for household food decisions (n = 499; 98.4%) and most respondents prepared food at home more than three times a week. Notably, few respondents were in action or maintenance stages of change for fruits or vegetables indicating that most respondents did not meet fruit and vegetable intake recommendations.

**Table 1:** Participants described and compared by eating competence categories

		Eating Competence Categories		
	**Total Sample^1^**	**Not Eating Competent^2^**	**Eating Competent^2^**	**p-Value^3^**
	**N = 507**	**N = 359**	**N = 148**	

Age	31.81 ± 6.98	31.84 ± 6.96	31.76 ± 7.06	.905
Race (n = 499)				.588
White	389 (78.0)	279 (79.0)	110 (75.3)	
Black	73 (14.6)	48 (13.6)	25 (17.1)	
Other	37 (7.4)	26 (7.4)	11 (7.5)	
Hispanic (n = 506)	31 (6.1)	18 (5.0)	13 (8.8)	.109
Education				.006
Some high school	56 (11.0)	39 (10.9)	17 (11.5)	
High school diploma/GED	179 (35.3)	118 (32.9)	61 (41.2)	
Some college	129 (25.4)	107 (29.8)	22 (14.9)	
≥ College degree	143(28.2)	95 (26.5)	48 (32.4)	
Parent	490 (96.6)	347 (96.7)	143 (96.6)	.984
Food Security Status^4 ^(n = 500)				.009
High	113 (22.6)	70 (19.8)	43 (29.5)	
Marginal	83 (16.6)	53 (15.0)	30 (20.5)	
Low	103 (20.6)	74 (20.9)	29 (19.9)	
Very Low	201 (40.2)	157 (44.4)	44 (30.1)	
Physically Active^5 ^(n = 506)	256 (50.6)	159 (44.4)	97 (65.5)	<.001
BMI (kg/m^2^) (n = 500)	30.41 ± 8.36	31.22 ± 8.44	28.44 ± 7.86	.001
Weight Categories (n = 500)				.025
Underweight (BMI < 18.5)	17 (3.4)	12 (3.4)	5 (3.4)	
Normal weight (BMI 18.5-24.9)	137 (27.4)	84 (23.7)	53 (36.3)	
Overweight (BMI 25.0-29.9)	118 (23.6)	84 (23.7)	34 (23.3)	
Obese (BMI ≥ 30.0)	228 (45.6)	174 (49.2)	54 (37.0)	
Weight dissatisfaction^6 ^(n = 506)	3.75 ± 1.23	3.94 ± 1.13	3.28 ± 1.33	<.001
**ecSI/LI**				
Total score	26.29 ± 9.70	21.36 ± 6.41	38.26 ± 4.53	<.001
Eating attitudes	9.52 ± 3.71	7.87 ± 2.99	13.53 ± 1.66	<.001
Internal regulation	5.45 ± 2.35	4.48 ± 1.96	7.80 ± 1.37	<.001
Food acceptance	3.93 ± 2.14	3.31 ± 1.84	5.44 ± 2.08	<.001
Contextual skills	7.39 ± 3.80	5.70 ± 2.81	11.49 ± 2.57	<.001
**TFEQ**				
Cognitive restraint	10.52 ± 2.12	10.45 ± 2.04	10.68 ± 2.30	.289
Disinhibition	7.74 ± 2.76	8.11 ± 2.73	6.83 ± 2.61	<.001
Feelings of hunger	6.69 ± 2.61	6.90 ± 2.68	6.16 ± 2.38	.004
**EDI-3**^7^				
Drive for thinness	13.19 ± 15.44	14.53 ± 15.87	9.93 ± 13.85	.002
Bulimia	26.03 ± 26.30	28.79 ± 26.57	19.33 ± 24.44	<.001
Body dissatisfaction	29.51 ± 25.08	32.89 ± 25.16	21.32 ± 22.98	<.001
Low self-esteem	21.13 ± 21.53	24.18 ± 21.99	13.75 ± 18.47	<.001
Personal alienation	23.88 ± 24.30	27.63 ± 25.34	14.80 ± 18.76	<.001
Interpersonal insecurity	32.37 ± 25.68	35.70 ± 25.48	24.31 ± 24.40	<.001
Interpersonal alienation	34.80 ± 27.04	38.94 ± 26.69	24.76 ± 25.26	<.001
Interoceptive deficits	17.30 ± 20.02	19.18 ± 20.58	12.76 ± 17.87	.001
Emotional dysregulation	31.13 ± 29.22	33.28 ± 28.84	25.94 ± 29.57	.010
Perfectionism	22.27 ± 22.09	21.60 ± 21.09	23.89 ± 24.35	.290
Asceticism	16.81 ± 20.51	17.62 ± 21.00	14.85 ± 19.20	.168
Maturity fears	46.17 ± 27.31	49.28 ± 27.24	38.62 ± 26.05	<.001
**Food Preference**				
Food like index^8^	7.85 ± 0.60	7.75 ± 0.59	8.10 ± 0.56	<.001
Food dislike index^9^	2.78 ± 0.83	2.76 ± 0.80	2.83 ± 0.90	.461
Foods never tried	1.88 ± 2.89	1.84 ± 2.87	2.00 ± 2.95	.561
**Fruit Stage of Change^10^**				
Action/Maintenance	209 (41.2)	130 (36.2)	79 (53.4)	<.001
**Vegetable Stage of change^10^**				
Action/Maintenance	62 (12.2)	33 (9.2)	29 (19.6)	.001
**EFNEP**^11^				
• Health locus of control	12.13 ± 2.05	12.01 ± 2.09	12.45 ± 1.93	.028
• Plans meals ahead of time	3.22 ± 1.06	3.04 ± 1.03	3.64 ± 1.00	<.001
• Plans meals to include all food groups	3.36 ± 1.00	3.21 ± 0.98	3.74 ± 0.95	<.001
• Makes a recipe successfully from scratch	3.53 ± 1.17	3.36 ± 1.18	3.95 ± 1.06	<.001
• Thinks about healthy food choices when deciding what to eat	3.43 ± 1.01	3.30 ± 0.99	3.74 ± 0.99	<.001
• Uses the "Nutrition Facts" on the food label to make food choices	2.70 ± 1.19	2.61 ± 1.14	2.93 ± 1.29	.006
• Eats out (including fast food)	2.56 ± 0.77	2.58 ± 0.79	2.52 ± 0.73	.412
• Shops with a grocery list	3.58 ± 1.19	3.48 ± 1.15	3.80 ± 1.25	.007
• Feels confident about managing money to make healthy food available	2.97 ± 1.18	2.87 ± 1.15	3.21 ± 1.21	.003
Prepares food at home > 3 times per week^12^	403 (79.5)	273 (76.0)	130 (87.8)	.003
Likes cooking^13 ^(n = 503)	227 (45.1)	142 (39.9)	85 (57.8)	<.001

Notes: Values are mean ± standard deviation or n (%); BMI = Body Mass Index; ecSI/LI = ecSatter Inventory for Low-Income; EDI-3 = Eating Disorders Inventory-3; EFNEP = Expanded Food and Nutrition Education Program; TFEQ = Three-Factor Eating Questionnaire; all measures are self-reported.

^1^N = 507 except where noted.

^2^Not eating competent = ecSILI < 32; eating competent = ecSILI ≥ 32.

^3^P-value for chi-square or t-test comparing eating competence categories.

^4^Reported for household.

^5^Survey item, "*Do you consider yourself a physically active person*? Response options - yes or no.

^6^Survey item, "*How satisfied are you with your current weight*?" 5-point response scale ranging from 1 = very satisfied to 5 = very unsatisfied.

^7^EDI-3 entries are normed percentiles.

^8^Food like index = Preference score of foods liked (denoted by preference selection of 5 to 9) divided by number of foods like. Possible range 5 though 9; higher score denotes greater preference.

^9^Food dislike index = preference score of foods disliked divided by number of foods disliked. Possible range 1 through 4; higher score denotes greater dislike.

^10^Computed scores collapsed to two categories - pre-action (precontemplation, contemplation, and preparation) vs. action/maintenance.

^11^For locus of control, range: 5 = external locus of control, 15 = internal locus of control. For all other EFNEP items, range: 1 = does not do, 5 = almost always does.

^12^Compared to preparing food at home ≤ 3 times per week.

^13^Survey item, "*How do you feel about cooking?*" Response options - *Likes cooking *compared to *Doesn't Like/Doesn't Mind Cooking*.

### Data Fidelity

Survey length prompted examination of response fidelity by inspection of responses to nearly similar items distributed throughout the web-based survey. Response fidelity was supported by several findings. For example, the item assessing weight dissatisfaction correlated with EDI-3 body dissatisfaction (r = 0.67, P < .001) and EDI-3 drive for thinness scores (r = 0.47; P < .001). Scores on the EDI-3 bulimia subscale correlated with the TFEQ disinhibition subscale scores (r = 0.58; P < .001). Scores on the US Adult Food Security Survey correlated to two single items referencing food security - an EFNEP item about frequency of running out of food before the end of the month (r = 0.73, P < .001) and an item directly referencing worry about having enough money for food (r = 0.59, P < .001).

### ecSI/LI Validation

Descriptive statistics for ecSI/LI total scale and four subscales (Eating Attitudes, Internal Regulation, Food Acceptance, and Contextual Skills) are presented in Table 1. Less than a third (29.2%) of respondents were eating competent. Analyses revealed internal consistency for all five scales (Cronbach's alpha coefficients = 0.90 for ecSI/LI total scale, 0.85 for Eating Attitudes, 0.79 for Internal Regulation, 0.64 for Food Acceptance, and 0.82 for Contextual Skills), as well as a wide range of ecSI/LI scores for respondents (range = 0 - 48), enabling psychometric analysis.

EC profiles were established by comparing dichotomized EC scores (Table 1). Compared to respondents without EC, respondents with EC were more physically active; reported greater incidence of normal weight; were less dissatisfied with their body weight and shape; expressed less tendency to overeat in response to external or emotional stimuli (TFEQ disinhibition scale); reported lower levels of psychosocial attributes related to disordered eating; had greater food acceptance; were more likely to meet fruit and vegetable intake recommendations; enjoyed cooking more; and practiced better food planning and resource management, including consideration for health and dietary variety.

ecSI/LI tertile scores (Table 2) endorsed the dichotomy-derived EC profiles, providing further validation evidence. Post hoc analyses revealed differences to be mostly between the lowest and highest EC tertiles. In addition, a majority of correlations between ecSI/LI scores and measures of eating behavior and attitudes met predetermined validity criteria in terms of direction and strength (Table 2). As predicted, ecSI/LI scores correlated inversely with cognitive and affective dimensions of eating behavior related to dieting and disordered eating, including weight and body dissatisfaction and psychological attributes related to disordered eating (low self-esteem, intra-and interpersonal alienation, and interpersonal insecurity). Total ecSI/LI scores correlated positively with Food Like Index and aspects of meal planning (planning ahead, planning to include all food groups, and thinking about healthy food choices), and shopping with a grocery list.

**Table 2:** ecSatter construct validation supported by ecSI/LI tertile comparison and measures of correlation

	**ecSI/LI Tertiles^1^**				
	
	**Lowest** **n = 175**	**Middle** **n = 163**	**Highest** **n = 169**	**p-value**^**2**^	***R***^**3**^
	
ecSI/LI total score	15.94 ± 4.18^a^	25.94 ± 2.69^b^	37.36 ± 4.87^c^	<.001	
Physically active^4 ^(n = 506)	68 (39.1)	76 (46.6)	112 (66.3)	<.001	
BMI (kg/m^2^) (n = 500)	32.15 ± 8.35^a^	30.72 ± 8.66^a^	28.33 ± 7.64^b^	<.001	-.17***
Weight Categories (n = 500)				.003	
Underweight (BMI < 18.5)	8 (4.7)	4 (2.5)	5 (3.0)		
Normal weight (BMI 18.5-24.9)	30 (17.6)	44 (27.0)	63 (37.7)		
Overweight (BMI 25.0-29.9)	39 (22.9)	42 (25.8)	37 (22.2)		
Obese (BMI ≥ 30)	93 (54.7)	73 (44.8)	62 (37.1)		
Weight dissatisfaction^5 ^(n = 506)	4.19 ± 1.02^a^	3.76 ± 1.18^b^	3.28 ± 1.30^c^	<.001	-.33***
**TFEQ**					
Cognitive restraint	10.41 ± 2.08	10.49 ± 2.01	10.66 ± 2.26	.551	.03
Disinhibition	8.49 ± 2.68^a^	7.78 ± 2.71^b^	6.93 ± 2.67^c^	<.001	-.25***
Feelings of hunger	6.98 ± 2.69^a^	6.87 ± 2.66	6.21 ± 2.43^b^	.014	-.14**
**EDI-3^6^**					
Drive for thinness	15.99 ± 14.29^a^	13.71 ± 17.97	9.78 ± 13.22^b^	.001	-.16***
Bulimia	33.79 ± 26.19^a^	24.04 ± 26.21^b^	19.91 ± 24.61^b^	<.001	-.24***
Body dissatisfaction	37.58 ± 23.82^a^	30.37 ± 26.22^b^	20.33 ± 22.13^c^	<.001	-.29***
Low self-esteem	28.02 ± 22.25^a^	21.31 ± 21.89^b^	13.83 ± 17.84^c^	<.001	-.28***
Personal alienation	31.90 ± 25.91^a^	24.64 ± 24.96^b^	14.85 ± 18.25^c^	<.001	-.29***
Interpersonal insecurity	40.23 ± 25.87^a^	33.18 ± 24.88^b^	23.46 ± 23.47^c^	<.001	-.28***
Interpersonal alienation	41.88 ± 26.65^a^	37.39 ± 26.52^a^	24.97 ± 25.14^b^	<.001	-.26***
Interoceptive deficits	22.93 ± 21.38^a^	15.85 ± 19.34^b^	12.88 ± 17.85^b^	<.001	-.21***
Emotional dysregulation	36.11 ± 28.86^a^	30.22 ± 28.24	26.85 ± 29.89^b^	.012	-.15**
Perfectionism	22.08 ± 21.33	20.56 ± 21.26	24.11 ± 23.60	.339	.04
Asceticism	18.34 ± 19.73	16.33 ± 21.36	15.69 ± 20.49	.456	-.06
Maturity fears	51.13 ± 26.52^a^	47.61 ± 28.59^a^	39.64 ± 25.67^b^	<.001	-.19***
**Food Preference**					
Food like index^7^	7.70 ± 0.63^a^	7.78 ± 0.56^a^	8.09 ± 0.55^b^	<.001	.31***
Food dislike index^8^	2.71 ± 0.81	2.83 ± 0.80	2.81 ± 0.89	.410	.03
Foods never tried	1.89 ± 2.95	1.82 ± 2.85	1.95 ± 2.88	.919	-.01
**Fruit Stage of Change^9^**					
Action/Maintenance	57 (32.6)	62 (38.0)	90 (53.3)	<.001	
**Vegetable Stage of Change^9^**					
Action/Maintenance	17 (9.7)	15 (9.2)	30 (17.8)	.027	
**EFNEP**^10^					
• Health locus of control	12.05 ± 2.04	11.95 ± 2.12	12.40 ± 1.99	.114	.10*
• Plans meals ahead of time	2.89 ± 1.01^a^	3.13 ± 1.02^a^	3.63 ± 1.00^b^	<.001	.33***
• Plans meals to include all food groups	3.10 ± 1.00^a^	3.28 ± 0.93^a^	3.71 ± 0.99^b^	<.001	.27***
• Makes a recipe successfully from scratch	3.26 ± 1.23^a^	3.45 ± 1.13^a^	3.88 ± 1.07^b^	<.001	.21***
• Thinks about healthy food choices when	3.20 ± 1.01^a^	3.34 ± 0.95^a^	3.75 ± 1.00^b^	<.001	.25***
deciding what to eat					
• Uses the "Nutrition Facts" on the food label	2.58 ± 1.11^a^	2.63 ± 1.17	2.90 ± 1.29^b^	.031	.15**
to make food choices					
• Eats out (including fast food)	2.55 ± 0.77	2.61 ± 0.80	2.53 ± 0.74	.600	-.04
• Shops with a grocery list	3.26 ± 1.14^a^	3.72 ± 1.10^b^	3.76 ± 1.25^b^	<.001	.19***
• Feels confident about managing money to	2.72 ± 1.15^a^	2.98 ± 1.11	3.21 ± 1.22^b^	<.001	.22***
make healthy food available					
Prepares food at home > 3 times per week^11^	127 (72.6)	130 (79.8)	146 (86.4)	.006	
Likes cooking^12 ^(n = 503)	60 (34.5)	75 (46.6)	92 (54.8)	.001	

Notes: Values are mean ± standard deviation or n (%); ^a, b, c ^denote statistically significant post hoc differences; BMI = Body Mass Index; ecSI/LI = ecSatter Inventory for Low-Income; EDI-3 = Eating Disorders Inventory-3; EFNEP = Expanded Food and Nutrition Education Program; TFEQ = Three-Factor Eating Questionnaire; all measures are self-reported.

^1^N = 507 except where noted.

^2^p-value for chi-square or analysis of variance comparing eating competence tertiles.

^3^Pearson correlation coefficients between total ecSI/LI score and row parameter; *p < .05, **p < .01, ***p < .001.

^4^Survey item, "*Do you consider yourself a physically active person*?" Response options - yes or no.

^5^Survey item, "*How satisfied are you with your current weight*?" 5-point response scale ranging from 1 = very satisfied to 5 = very unsatisfied.

^6^EDI-3 entries are normed percentiles.

^7^Food like index = Preference score of foods liked (denoted by preference selection of 5 to 9) divided by number of foods like. Possible range 5 though 9; higher score denotes greater preference.

^8^Food dislike index = preference score of foods disliked divided by number of foods disliked. Possible range 1 through 4; higher score denotes greater dislike.

^9^Computed scores collapsed to two categories - pre-action (precontemplation, contemplation, and preparation) vs. action/maintenance.

^10^For locus of control, range: 5 = external locus of control, 15 = internal locus of control. For all other EFNEP items, range: 1 = does not do, 5 = almost always does.

^11^Compared to preparing food at home ≤ 3 times per week.

^12^Survey item, "*How do you feel about cooking?*" Response options - *Likes cooking *compared to *Doesn't Like/Doesn't Mind Cooking*.

ecSI/LI subscales (Eating Attitudes, Food Acceptance, Internal Regulation, and Contextual Skills) correlated with measures of cognitive behavior, disordered eating, food management, and food preference suggesting support for construct validity. Eating Attitudes subscale scores were inversely associated with dietary disinhibition (TFEQ disinhibition scale r = -0.27), weight dissatisfaction (r = -0.36), and EDI-3 scales that indicate disordered eating (drive for thinness; r = -0.26; bulimia r = -0.31; body dissatisfaction r = -0.30; low self-esteem r = -0.26; personal alienation r = -0.25; interpersonal insecurity r = -0.25, interoceptive deficits r = -0.21, and maturity fears r = -0.20). Internal Regulation subscale scores were inversely associated with disinhibition (r = -0.24) and weight dissatisfaction (r = -0.27) as well as several EDI-3 subscales showing an inverse association between internal regulation and disordered eating (drive for thinness r = -0.20, body dissatisfaction r = -0.25, and bulimia r = -0.24). Food Acceptance subscale scores were positively associated with food preference (Food Like Index r = 0.23) and inversely associated with number of foods that have never been tried (r = -0.21). In addition, Food Acceptance subscale scores were positively associated with several indicators that food is considered in meal planning, e.g. making a recipe successfully from scratch (r = 0.26), thinking about healthy food choices when making food decisions (r = 0.32), and using Nutrition Facts labels (r = 0.28). Contextual skills positively correlated with nearly all measures of food management practices (e.g., planning meals r = 0.43, including all food groups in meals r = 0.33, thinking about healthful food choices when planning what to eat r = 0.33, using Nutrition Facts labels r = 0.26, and confidence in managing money for food r = 0.30).

## Discussion

ecSatter construct validity was affirmed by comparison of ecSI/LI responses to outcomes of validated measures of cognitive and affective eating behavior, food preference and practice, and food preparation. Most correlations and differences between eating competent and non-eating competent categories, or among EC tertiles, were compatible with hypothesized relationships established with the validation of the ecSI [22]. As anticipated, EC was related to being physically active, action/maintenance stages of change for fruit and vegetable intake, greater food preference and food preparation and planning skills, and normal BMI, and lower levels of dietary disinhibition, weight dissatisfaction, and body image dissatisfaction. EC was also compatible with an absence of disordered eating as measured by the EDI-3 [29]. Thus, use of ecSI/LI to measure EC in low-income women was affirmed.

Cognitive restraint (as measured by the TFEQ scale) was not related to EC in the present study. Absence of association between EC and the TFEQ cognitive restraint scale may be reflective of sample differences between our sample and that of Lohse et al [22]. Compared to the original validation sample, our sample reported higher levels of dietary restraint (10.52 ± 2.12 vs 9.0 ± 5.0). Lohse et al's [22] sample included both males and females with 93% having education beyond high school compared to only 54% in the present study. Females in our sample were younger (mean age 31.81 ± 7.0 years vs 36.2 ± 13.4) and had a greater mean BMI (30.4 ± 8.4 vs 25.7 ± 6.1) compared to Lohse et al [22]. Our sample showed less economic diversity; 64% of our sample reported being sometimes, often, or always "worried about having enough money food" compared to 23% of their sample. In addition, Contento et al [38] reported a similar level of cognitive restraint (10.54 ± 4.78) for a sample of primarily low-income Latino mothers. Thus, differences in dietary restraint between the present sample and the original validation sample may reflect eating attitudes and behaviors attributable to being low-income or having excess weight or both.

Dietary restraint is widely regarded as control of food intake to prevent weight gain or promote weight loss. However, insufficient or unreliable food availability may inadvertently manifest as restraint. In fact, 61% of our sample reported low or very low levels of food security, which is characterized by reduction in food intake and inconsistent eating patterns for one or more members of a household [39]. Unreliable availability of food mimics food restriction, possibly creating a tendency for eating greater amounts when food is plentiful and disrupting self-reliance on internal regulation of food intake [40,41]. Alternatively, participants may be attempting weight control through intentional food restriction and reacting to those efforts by binging. Dietary restraint is often linked to disinhibition, such that excessive weight concern or strict control of food intake results in loss of control of eating in the presence of various stressors, such as emotional or situational stimuli [28,42,43]. Dietary disinhibition is considered a more precise measure of disturbed eating behaviors than cognitive restraint [44,45]. In the present study, disinhibition decreased as EC increased, even in the absence of changes in cognitive restraint. Several researchers have noted a relationship between emotional eating, overeating, and overweight/obesity among low-income women [40,46-49]. Developing EC, including learning to eat in response to internal cues of hunger and satiety, may reduce the likelihood of eating in response to emotional cues [20].

This study has a number of strengths and weaknesses. Participants were randomly selected from a defined population of low-income females living in households with children under the age of 18. Females of child-bearing age play a central role in family food purchases and preparation and are key targets of federal food assistance programs. Nearly all study participants were mothers (97%) reporting sole or shared responsibility for household food decisions (98%). However, results may not generalize to males, different age groups or across ethnicities or other geographical regions. Previous research indicates that young adult males have dissimilar profiles of eating behavior, which may be reflective of gender-specific weight goals [23]. In addition, online administration of the study required that participants have access to the internet, which may not have been available to some who were invited to participate. This may raise concerns regarding sample representativeness because low-income woman without internet access may differ systematically from our respondents. However, recent findings suggest that the internet is available to low-income audiences [25,50,51]. Surveys were administered in English, which prevented individuals unable to read English from participating.

Another limitation is our assumption that respondents considered choices in the present tense. Schwarzer [52] has developed a theory of health behavior change, the Health Action Process Approach (HAPA) that has interesting implications for how our results are viewed. According to this model, behavior change is a self-regulatory process divided into motivation and volition (pursuit of goals). The volitional phase is in turn composed of Intenders (i.e. those who have not translated their intentions into action) and those who have made that transition, i.e. Actors. A limitation of our study is that we assumed participants answered the *present tense *ecSI/LI items correctly categorizing their perceived volitions as intentions or actions. For example, a participant may truly perceive they are on the verge of trusting themselves to eat enough for them and answer as though they fully express that behavior. Thus, our validation assumes respondents were Actors, not Intenders and our study did not include distinguishing activities or items. Interventions are not as necessary for Actors and have characteristics quite apart from those needed by Intenders. This possibility, coupled with the fact that only 29% of the current sample was found to be eating competent, supports development and implementation of interventions to enhance EC.

## Conclusions

Findings from this study suggest that ecSI/LI is a valid measure of EC. Messick [53] marks validity as "... an evolving property and validation a continuing process." This study extended the exoneration of ecSI/LI from having construct-irrelevant variance due to difficulty for low-income audiences [27] by providing an evidential basis for test interpretation (i.e. construct validity) in a low-income sample. Availability of a valid measure of EC will benefit assessment of interventions designed to engender EC in low-income audiences and enable progression of the validation process to considerations of values and social consequences of eating competence education.

## Competing interests

The authors declare that they have no competing interests.

## Authors' contributions

Both authors conceived the study and contributed to the study design, statistical analysis, interpretation of the results, and manuscript preparation. JSK coordinated the study and data collection. Both authors read and approved the final manuscript.
